# Implementation of Safe Sleep Practice Recommendations for Infants in Inpatient Wards

**DOI:** 10.7759/cureus.11155

**Published:** 2020-10-25

**Authors:** Mrouge Sobaihi, Maysaa A Banjari, Turki S Alahmadi

**Affiliations:** 1 Pediatrics, King Faisal Specialist Hospital and Research Centre, Jeddah, SAU; 2 Pediatrics, King Abdulaziz University, Jeddah, SAU; 3 Pediatrics, King Abdulaziz University, Rabigh, SAU

**Keywords:** sudden infant death syndrome, sleep practice, infant sleep, sids, sleep position

## Abstract

Background

Sudden infant death syndrome (SIDS) is defined as the sudden unexpected death of an infant, even after conducting thorough investigations and autopsy. SIDS is related to several factors, such as baby’s position and presence of pillows, blankets and objects in the crib. The implementation of safe sleep recommendations in the inpatient setting is unknown and there is a scarcity of available data.

Methods

This was an observational, cross-sectional study that was conducted at King Abdulaziz University Hospital, Jeddah, Saudi Arabia. All infants less than one year of age were considered after fulfilling the inclusion criteria. A checklist was developed in alignment with the latest American Association of Pediatrics (AAP) recommendations and an an independent observer was trained on how to evaluate and record the various components of the checklist.

Results

One hundred and two patients were enrolled in this study. The mean age of participants was 18.85 weeks. Asleep infants were found to be mostly placed in their cribs (71.4%), on their back (81%). Among the sleeping infants, 46% of them were swaddled at the time of data collection. Blankets were present in 79.4% of the cases, and loose sheets in more than half of the cases. Pillows were seen in 42.2% of the beds. No bumper pads were present in any of the beds.

Conclusion

This observational study highlights the importance of increasing awareness about safe sleep practices. Not only is it important for ensuring patient safety during admissions but also to send an important message to caregivers through role-modeling. Further studies are required to examine the barriers to implementing recommended safe sleep practices both within institutions that care for infants as well as among parents and caregivers.

## Introduction

When an infant less than one year of age dies during sleep for any reason, it is described as a sleep-related infant death (SRID) [1]. Sleep-related infant deaths can occur due to various causes including infectious, cardiac, allergic and metabolic causes. When the cause of death remains unknown after thorough investigations and scene assessment, the sleep-related death is then classified as sudden infant death syndrome (SIDS) [2, 3].

In 1992, the American Academy of Pediatrics (AAP) released its first guidelines to address SIDS and recommended safer sleep practices mainly highlighting the importance of placing the infant on his/her back during sleep [4]. This was based on the recognition that the prone position significantly increased the risk of SIDS. After the implementation of the AAP recommendations, a significant decline in the prevalence of SIDS occurred [5].

With time, it was acknowledged that other factors such as strangulation and suffocation also played a role in sleep-related infant deaths [6,7]. This lead to the increased use of the term “sleep-related deaths” rather than solely SIDS in order to encompass all etiologies. The recommendations for safe sleep practices were modified to address additional important safety practices including but not limited to discouraging bed-sharing, soft mattresses and loose blankets, soft toys, and pillows [8].

The incidence of sleep-related infant death is not currently accurately reported in Saudi Arabia. A study conducted in 1995 estimated the rate of SIDS to be 1.3 per 1000 live births. However, the study took place in Qatif city and thus may not represent national statistics. Furthermore, no autopsies were performed [9]. Two other retrospective single-centre studies reviewed infant mortalities in general but did not specifically address sleep-related infant deaths [10, 11]. Moreover, one of the two studies had excluded infants who arrived dead to the hospital. Furthermore, there are no local researches on parents' knowledge of safe sleep habits, This points towards a scarcity in the available data. Nonetheless, there is enough evidence worldwide to necessitate the implementation of safe sleep practices.

To assess adherence to safe sleep guidelines, we believe that our 70-bed pediatric inpatient units at King Abdulaziz University Hospital (KAUH) represent a good starting point to examine. Through our preliminary study, we strive to examine the current infant sleep practices within various inpatient units will aid in identifying any gaps and help focus future interventional strategies and hospital policy development. The study findings will complement international studies targeting similar subjects with consideration of specific cultural contexts and variations. 

## Materials and methods

This study has been reviewed and approved by the institutional review board at King Abdulaziz University.

It is an observational cross-sectional study to look at the current sleep practices being implemented in the 70-bed pediatric inpatient units at King Abdulaziz University Hospital (KAUH), which is a tertiary hospital located in an urban area of Jeddah, Saudi Arabia. Informed consent was obtained from all caregivers.

All infants less than one year of age were considered. Exclusion criteria were infants in the neonatal or intensive care units or those who were on a mechanical ventilator or on respiratory assistance of any kind as well as patients with diagnosed upper airway anomalies.

A checklist was developed in alignment with the latest AAP recommendations (Appendix). An independent observer was trained on how to evaluate and record the various components of the checklist. The main categories observed were related to sleep position, sleep surface, the presence of any soft toys, pillows or blankets, and pacifier use. Data was entered in SPSS (Statistical Package for the Social Sciences) version 20 (IBM Corp, Armonk, USA) for analysis.

## Results

**Figure 1:**
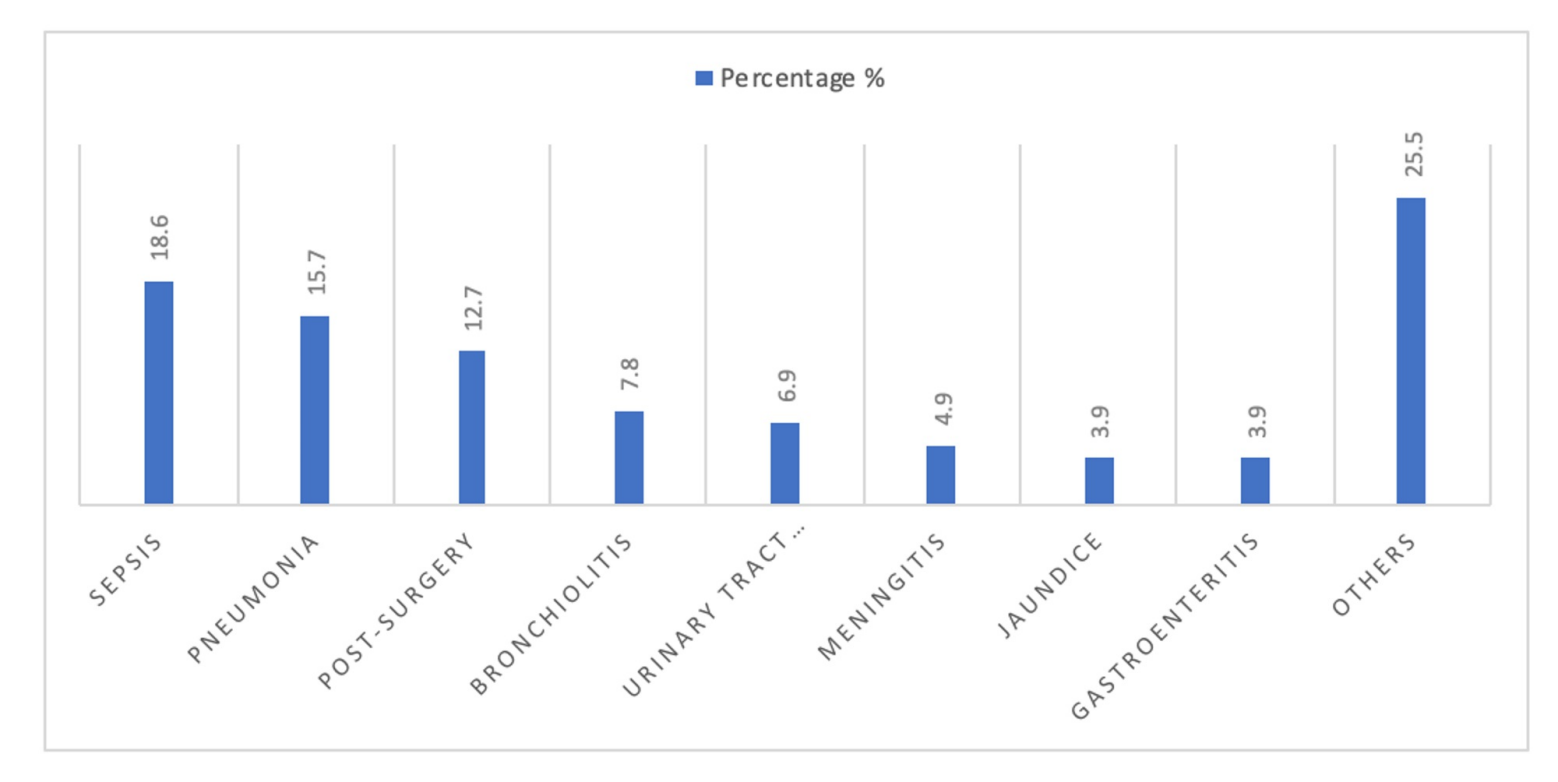
Admission diagnoses

One hundred and two patients were enrolled in this study. The mean age of participants was 18.85 weeks and (64.7 %) of them were males. The list of their reasons for admission is detailed in Figure 1.

Infants were asleep in 61.8% of encounters (n=63). Out of those, 50 (79.3%) were in their cribs, 12 (19%) sharing a bed with an adult, and one infant was sleeping in the baby seat. Fifty-one (81%) infants were placed on their back, 12 (19%) on their side, and none were found in the prone position. Cloth swaddles were used 46% of encounters. More details are present in Table 1.

**Table 1:** Location and position of sleeping infants (n=63)

	N (%)
Asleep Infant is placed in:	
Crib	50 (79.3)
Bed with an adult	12 (19)
Baby seat	1 (1.6)
Position of the infant	
Back	51 (81)
Side	12 (19)
Prone	0 (0)
Infant is swaddled	29 (46)

With regard to sleep surfaces, all mattresses were firm or hard and no soft mattresses were used. Blankets were present inside the crib in 79.4% of the time, loose sheets in 55.9%, and pillows used in 42.2% of encounters. No bumper pads were placed in any of the beds. Further details are mentioned in Table 2.

**Table 2:** Crib environment (all infants = 102)

	N (%)
Found in the crib	
Blanket	81 (79.4)
Loose sheet	57 (55.9)
Pillow	43 (42.2)
Bumper pads	0 (0)
Soft toys	6 (5.9)
Electrical wires	30 (29.4)
Crib is away from the window	102 (100)
Mattress Firmness	
Soft	0 (0)
Firm	96 (94.1)
Hard	6 (5.9)
Space between crib and mattress	
Two fingers or less	100 (98%)
More than two fingers	2 (2)

## Discussion

Despite the development of thorough guidelines and initial decline in SIRDS rates, there has been a plateau over the last 15 years [12]. The cause of this plateau is not completely understood and multiple factors likely play a role including adherence to guidelines. Role modeling safe sleep practices is essential for propagating the right practices within society.

Implementation of safe sleep practices requires both an increased awareness and acceptance ‘buy-in’ from caregivers. Hospital staff and healthcare providers play an essential role in increasing this awareness and implementing safe sleep practices. In order for the education and principles to be believable, hospitals need to role model adherence to safe sleep practices within their institution. It is easy to imagine the detrimental effects that can happen by ‘setting a bad example’. In addition, evidence has shown that the sleep practices modeled in the hospital before discharge, influence the habits that parents adopt at home in both positive and negative directions [13,14].

This study indicates that adherence to ideal safe sleep habits in in-patient settings is suboptimal. Although parts of the recommendations were adhered to more often than others, 100% adherence could only be observed in four out of 63 sleeping infants (6.3%). This is similar to what was found in a study conducted in the United States, which reported that 4.9% (13/264) of infants had a safe sleep environment during their hospital stay [15].

Out of all safe sleep recommendations, placing the infant on his/her back (position) was the most followed. None of the sleeping infants was found prone and the majority (81%) were on their backs. Nevertheless, 19% were still sleeping on their sides. This observation is similar to what was seen in Arkansas Children Hospital, where 72% of sleeping infants were placed supine [16]. Furthermore, the majority (79.3%) of infants were placed inside their cribs to sleep. However, 19% of the infants shared a bed with their caregivers despite the presence of a crib in the room. It was observed that the mother preferred this sleeping arrangement to have easier access to the infant at night.

It was also noticed 46% of sleeping infants were swaddled. It is debatable whether swaddling influences the incidence of SIDS and there is insufficient evidence for or against it [17, 18]. However, there is some evidence that using swaddling may be dangerous if the infant rolls to the prone position. This is why it was recommended in the new AAP guidelines that swaddles should not be used if the infant starts to show signs of rolling attempts. If used prior to that developmental milestone, care needs to be taken to ensure they are not loose and should be tucked tightly around the chest [1]. In addition, considerations need to be made in order to avoid exacerbating hip dysplasia [19].

We found that keeping the infant crib empty and free of hazards was the most identified challenge. Blankets and loose sheet were used at a very high rate (79-4% and 55.9% respectively) despite the fact that they’re not provided regularly by the staff. This indicates that caregivers are bringing them from outside. Using sleeping bags have been coming into practice in some institution [20]. It was used to mitigate this risk and as an alternative for the blankets. On the other hand, there were no clearly identified hazards within the reach of the infant. However, even when the crib its self was almost in line with the AAP guidelines, the surrounding areas were frequently neglected. Frequently cables (through an electrical extension) and phone chargers were within the baby’s reach.

This is a very important subject to address as practices modeled in the healthcare setting are learned and carried on at home [13,14,21]. Since adherence in the inpatient setting was suboptimal, there is an identified need to assess the barriers that prevented full implementation of safety guidelines. These barriers are likely to be multifactorial and to stem from both health care providers as well as parents and caregivers.

Hypothesized challenges for health care providers may include insufficient awareness of the guidelines including misconceptions about certain definitions, e.g., co-sleeping versus bed-sharing, certain behaviors such as reluctance to alter incorrect habits when observed, or lack of time to adequately audit the sleep environment. A study done in Brazil on paediatricians’ knowledge of SIDS risk factors revealed that only 66% of them identified bedsharing as a risk factor [22]. 

Parent and caregiver barriers may include unawareness of the guidelines as well as cultural values and beliefs such as swaddling and keeping the baby away from air conditioner breeze by covering the crib with a blanket or covering the baby’s face. Parents were using the baby crib to hold their extra belongings for convenience.

It is well known that interventions to improve sleep practices should be multi-layered and should be tackled from different angles. Addressing cultures, behaviors, healthcare provider knowledge and attitudes, and regulations have been promoted as the levels were interventions should be considered simultaneously [23]. 

Limitations

This study has several limitations, firstly, patients were observed during the daytime, so variations between day and night practices were not addressed. Secondly, caregivers’ knowledge and its relation to the sleep environment practised were not assessed, Thirdly, the knowledge and practice of healthcare providers were not assessed. The authors believe that interview-based qualitative research could yield more accurate results and provide a better understanding of caregivers and staff knowledge and misbeliefs on safe sleep practices for infants.

## Conclusions

Our study highlights the importance of increasing awareness of safe sleep practices. The hospital setting is an important environment for the initiation of safe sleep habits. Not only is it important for ensuring patient safety during admissions but also to send an important message to caregivers through role-modeling. Further studies are required to examine the barriers to implementing 100% safe sleep practices both within institutions that care for infants as well as among parents and caregivers. These studies would help develop interventional strategies and guide policy development about this important subject.

## Appendices

Table 3, Table 4, and the text below present the checklist that was developed in alignment with the latest AAP recommendations and was used to collect the data by observing the participants.

**Table 3:** Exclusion criteria of the study If any of the above questions is answered yes, don’t proceed with the form.

	Yes	No
Is the baby more than 1 year?		
Tracheostomy		
Ventilated (Invasive or non- invasive)		
Upper airway anomaly		
Spinal surgery		
He is critically sick		
He is an ICU patient		
Awake		

**Table 4:** Sleeping characteristics

1) Where is the infant sleeping?								
Crib			Bed alone				Bed with an adult	
Couch			Stroller				Baby seat	
Swing			Infant carriers				Infant sling	
2) In which position is the infant sleeping?								
Back (100% on the back)			Side				Prone	
3) The crib is near the window			Yes				No	
4) Do you see any of the following inside the crib?								
Loose sheet	Pillow	Blanket		Pillow like toys	Electrical wires	Bumper pads		soft toys

Time of assessment: ______ AM/PM Age: Sex:

Diagnosis:

Caregiver received education about safe sleep practices? 

## References

[1] Task Force on Sudden Infant Death Syndrome (2016). SIDS and other sleep-related infant deaths: updated 2016 recommendations for a safe infant sleeping environment. Pediatrics.

[2] Willinger M, James LS, Catz C (1991). Defining the sudden infant death syndrome (SIDS): deliberations of an expert panel convened by the National Institute of Child Health and Human Development. Pediatr Pathol.

[3] Beckwith JB (2003). Defining the sudden infant death syndrome. Arch Pediatr Adolesc Med.

[4] (1992). American Academy of Pediatrics AAP Task Force on Infant Positioning and SIDS: positioning and SIDS. Pediatrics.

[5] Gibson E, Fleming N, Fleming D (1998). Sudden infant death syndrome rates subsequent to the American Academy of Pediatrics supine sleep position. Med Care.

[6] Shapiro-Mendoza CK, Kimball M, Tomashek K, Anderson RA, Blanding S (2009). US infant mortality trends attributable to accidental suffocation and strangulation in bed from 1984 through 2004: are rates increasing?. Pediatrics.

[7] Pasquale-Styles MA, Tackitt PL, Schmidt CJ (2007). Infant death scene investigation and the assessment of potential risk factors for asphyxia: a review of 209 sudden unexpected infant deaths. J Forensic Sci.

[8] Task Force on Sudden Infant Death Syndrome (2011). SIDS and other sleep-related infant deaths: expansion of recommendations for a safe infant sleeping environment. Pediatrics.

[9] Srair HA, Owa JA, Aman HA (1995). Cause-specific infant mortality rate in Qatif area, eastern province, Saudi Arabia. Ann Saudi Med.

[10] Nofal HK, Abdulmohsen MF, Khamis AH (2011). Incidence and causes of sudden death in a university hospital in eastern Saudi Arabia. East Mediterr Health J.

[11] Jelly AE, Warnasuriya N, Pejaver RK (1998). A study of infant deaths in a regional hospital in Saudi Arabia. Saudi Med J.

[12] Hwang SS, Corwin MJ (2017). Safe infant sleep practices: parental engagement, education, and behavior change. Pediatr Ann.

[13] Colson ER, Bergman DM, Shapiro E, Leventhal JH (2001). Position for newborn sleep: associations with parents' perceptions of their nursery experience. Birth.

[14] Colson ER, Joslin SC (2002). Changing nursery practice gets inner-city infants in the supine position for sleep. Arch Pediatr Adolesc Med.

[15] Kuhlmann S, Ahlers-Schmidt C, Lukasiewicz G, Truong TM (2016). Interventions to improve safe sleep among hospitalized infants at eight children's hospitals. Hosp Pediatr.

[16] Rowe A, Sisterhen L, Mallard E (2016). Integrating safe sleep practices into a pediatric hospital: outcomes of a quality improvement project. J Pediatr Nurs.

[17] Thach BT (2009). Does swaddling decrease or increase the risk for sudden infant death syndrome?. J Pediatr.

[18] McDonnell E, Moon RY (2014). Infant deaths and injuries associated with wearable blankets, swaddle wraps, and swaddling. J Pediatr.

[19] Ömeroğlu H, Akceylan A, Köse N (2019). Associations between risk factors and developmental dysplasia of the hip and ultrasonographic hip type: a retrospective case control study. J Child Orthop.

[20] Voos K, Terreros A, Larimore P, Leick-Rude MK, Park N (2015). Implementing safe sleep practices in a neonatal intensive care unit. J Matern Fetal Neonatal Med.

[21] Brenner R, Simons-Morton B, Bhaskar B (1998). Prevalence and predictors of the prone sleep position among inner-city infants. JAMA.

[22] Maestri RN, Nunes ML (2016). The uptake of safe infant sleep practices by Brazilian pediatricians: a nationwide cross-sectional survey. Sleep Med.

[23] Moon RY, Hauck FR, Colson ER (2016). Safe infant sleep interventions: what is the evidence for successful behavior change?. Curr Pediatr Rev.

